# Crystal structure of potassium (1*R*)-d-ribit-1-yl­sulfonate

**DOI:** 10.1107/S1600536814022685

**Published:** 2014-10-24

**Authors:** Alan H. Haines, David L. Hughes

**Affiliations:** a School of Chemistry, University of East Anglia, Norwich NR4 7TJ, England

**Keywords:** crystal structure, d-ribose bis­ulfite adduct, potassium hydrogen sulfite, potassium metabisulfite

## Abstract

The title compound, K^+^·C_5_H_11_O_8_S^−^ [systematic name: potassium (1*R*,2*R*,3*R*,4*R*)-1,2,3,4,5-penta­hydroxy­pentane-1-sulfonate], formed by reaction of d-ribose with potassium hydrogen sulfite in water, crystallizes as colourless plates. The anion has an open-chain structure in which the S atom and the C atoms of the sugar chain, excepting that of the hy­droxy­methyl group, form an essentially all-*trans* chain; the C atom of the hy­droxy­methyl group lies in a *gauche* relationship with the three contiguous C atoms. Through complex cation coordination (through seven oxygen atoms of six different anions) and inter­molecular O—H⋯O hydrogen bonding, a three-dimensional bonding network exists in the crystal structure.

## Abstract 2

The anion of potassium (1*R*)-d-ribit-1-yl­sulfonate has an open-chain structure with the potassium cation seven-coordinated in an approximately penta­gonal–bipyramidal coordination environment by six different anions through K—O coordinate bonds.

## Chemical context   

Addition compounds formed between carbonyl compounds and the bis­ulfite anion have found use in purification of liquid aldehydes when, as is often the case, the adduct is crystalline, in facilitating cyano­hydrin formation, and also in conferring required water solubility to certain hydro­phobic compounds (Clayden *et al.*, 2012 ▶). Less well known is the fact that aldoses, despite existing preferentially in the hemiacetal form, can react with the bis­ulfite anion to give open-chain adducts which, as chiral hy­droxy­sulfonic acids, have potentially useful but largely unexplored applications in synthesis. The know­ledge of such compounds was initially centred on the their possible role in the stabilization of food stuffs (Gehman & Osman, 1954 ▶) (note: nearly all wines are labelled ‘contains sulfites’) and evidence for their acyclic nature was first provided by Ingles (1959 ▶), who prepared such adducts from d-glucose, d-galactose, d-mannose, l-arabinose and l-rhamnose. However, conclusive proof for their acyclic structure awaited X-ray studies, initially by Cole *et al.* (2001 ▶) who reported the crystal structures of d-glucose- and d-mannose-derived potassium sulfonates, and later we studied the sodium sulfon­ate derived from d-glucose (Haines & Hughes, 2012 ▶) and the potassium sulfonate from d-galactose (Haines & Hughes, 2010 ▶) by X-ray crystallography. The crystal structure of the potassium bis­ulfite adduct of de­hydro-l-ascorbic acid, first prepared by Ingles (1959 ▶), has also been reported (Haines & Hughes, 2013 ▶).

*C*-Sulfonic acid derivatives of carbohydrates have been prepared at non-glycosidic atoms by the radical-mediated addition of the bis­ulfite ion to methyl 6-de­oxy­hexo­pyran­osid-5-enes (*e.g.* in the synthesis of 6-sulfoquinovose; Lehmann & Benson, 1964 ▶), by tri­fluoro­methane­sulfonate-mediated nucleo­philic displacement reactions with the bis­ulfite ion (Lipták *et al.*, 2004 ▶) or by oxidation of a thio­acetyl substituent on a protected glycose (Lipták *et al.*, 2004 ▶). Although oxidation of C1-thio­esters of protected aldoses affords a route to C1-sulfonic acids, the facile preparation of the bis­ulfite adducts of certain aldoses provides an attractive route to chiral hy­droxy­sulfonic acids, which merit further exploration as possible synthetic inter­mediates.
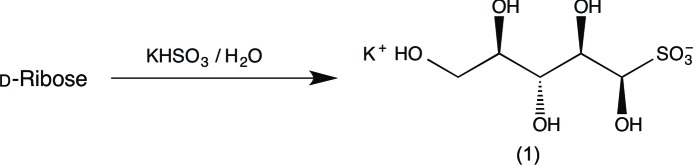


Preparation of aldose adducts requires reaction at high concentrations, with the bis­ulfite anion produced *in situ* by hydrolysis of the corresponding metabisulfite. Obtaining suitable material for X-ray crystallography is not always straightforward, either in the initiation of crystallization or in isolating crystals of suitable quality. We report here the preparation in crystalline form of the hitherto unknown potassium bis­ulfite adduct from d-ribose, (1), and its solid-state structure.

## Structural commentary   

**Figure 1:**
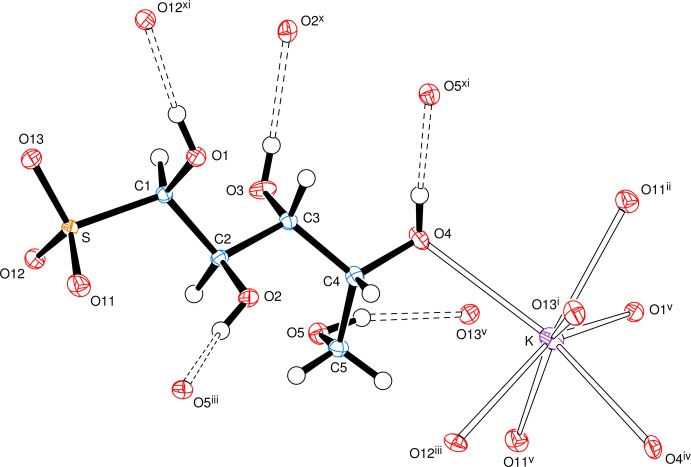
View of a mol­ecule of potassium (1*R*)-d-ribit-1-yl­sulfonate, indicating the atom-numbering scheme, showing the hydrogen bonds (dashed lines) from the anion and the coordination pattern around the potassium cation. Displacement ellipsoids are drawn at the 50% probability level. Symmetry codes: (i) −*x*, *y* + 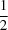, −*z* + 1; (ii) *x* − 1, *y*, *z* − 1; (iii) −*x* + 1, *y* + 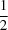, −*z* + 1; (iv) −*x*, *y* + 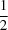, −*z*; (v) *x*, *y*, *z* − 1; (vi) *x*, *y*, *z* + 1; (vii) −*x*, *y* − 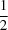, −*z*; (viii) *x* + 1, *y*, *z* + 1; (ix) −*x* + 1, *y* − 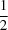, −*z* + 1; (x) −*x*, *y* − 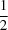, −*z* + 1; (xi) *x* − 1, *y*, *z*; (xii) *x* + 1, *y*, *z*.

**Figure 2:**
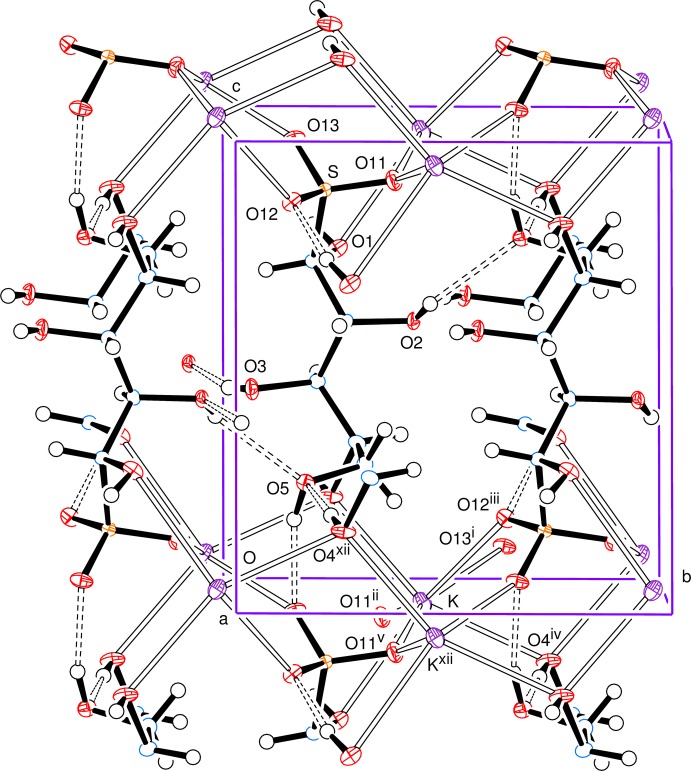
View approximately along the *a* axis, showing the hydrogen-bonding contacts and all the K—O coordination bonds. Symmetry codes as in Fig. 1 ▶.

The anion has an open-chain structure in which carbons C1 to C4 together with O4, S and O13 form an essentially all-*trans* chain (Fig. 1 ▶), with the newly formed chiral centre at C1 having the *R*-configuration. The systematic name for the salt is potassium (1*R*,2*R*,3*R*,4*R*)-1,2,3,4,5-penta­hydroxy­pentane-1-sulfonate. The torsion angle C2—C3—C4—C5 is indicative of a *gauche* conformation with C5 pointing out of the all-*trans* chain. All of the hydroxyl groups form O—H⋯O hydrogen bonds and all, except for the hydrogen bond from O2, have short H⋯O distances with O—H⋯O angles not far from linear (Table 1 ▶); the O2 hydrogen bond is towards the upper limit in terms of H⋯O distance with an angle of 132 (2)° at H2*O*. The potassium ions are seven-coordinate with K—O bonds to six separate anions; the K—O bond lengths lie in the range of 2.7383 (10) to 3.0085 (11) and are arranged in an approximately penta­gonal–bipyramidal form with O4 and O4^iv^ as the apical atoms. This is shown in Fig. 2 ▶, a view approximately along the *a* axis, indicating the hydrogen-bonding contacts and the K—O coordinate bonds. Potassium ions can show various coordination numbers in related coordination environments: in the d-galactose bis­ulfite (Haines & Hughes, 2010 ▶), d-glucose bis­ulfite (Cole *et al.* 2001 ▶; Haines & Hughes, 2012 ▶) and de­hydro-l-ascorbic acid bis­ulfite (Haines & Hughes, 2013 ▶) adducts, the potassium ion is, respectively, six-, seven-, and eight-coordinate.

**Figure 3:**
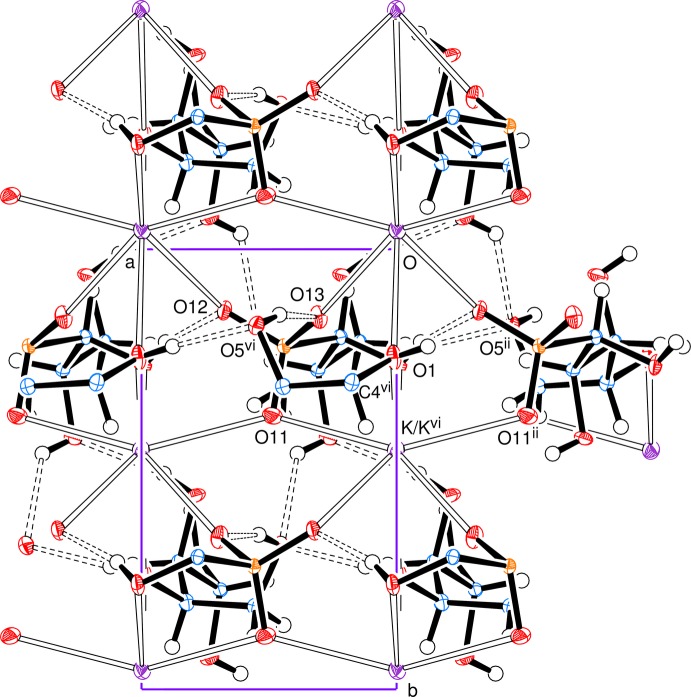
View along the *c* axis, showing the parallel alignment of the open-chain ions and the inter­ionic inter­actions. Symmetry codes as in Fig. 1 ▶.

**Figure 4:**
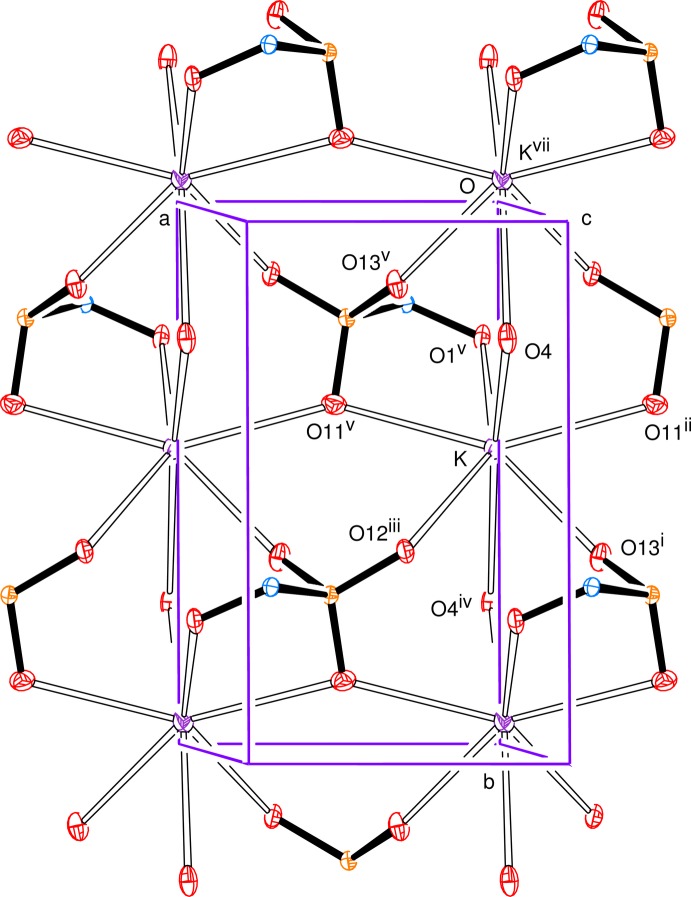
A section parallel to the *ab* plane around *z* = 0, showing the linking of the potassium ions in that plane; the connections are made through coordination bonds involving the sulfonate groups and the hydroxyl groups of O1 and O4. Symmetry codes as in Fig. 1 ▶.

Fig. 3 ▶, a view down the *c* axis, indicates the parallel alignment of the open-chain ions and Fig. 4 ▶ illustrates a section parallel to the *ab* plane showing the linking of the potassium ions in that plane.

High-resolution mass spectrometry in negative-ion mode identified the anion at *m*/*z* 231.0187 but the base peak was at *m/z* 213.0082, representing loss of water from the parent ion. A large peak was also observed at 299.0987 for C_10_H_19_O_10_, which corresponds to the ion of the product formed by reaction between (1) and d-ribose with displacement of potassium bis­ulfite; in the aqueous solution used for MS analysis, some decomposition of (1) to afford d-ribose undoubtedly occurs and this is supported by NMR data on the aqueous solution reported below.

The ^1^H NMR spectrum of (1) in D_2_O indicates considerable stability of the adduct in aqueous solution, with the species α-furan­ose, β-furan­ose, β-pyran­ose, α-pyran­ose, and bis­ulfite adduct, identified by their H-1 resonances, present in the % ratios of 3.6:6.2:10.9:5.1:74.2, which changed only marginally after 18 days. A complete assignment of the spectrum for (1) and consideration of derived coupling constants indicated overall similarity of the conformation in the crystalline state and in solution. Notably, *J*_1,2_ was close to zero and assuming Newman projection angles of 120° and using measured torsional angles, a Karplus relationship suggests a value of about 0.3 Hz. The value *J*_2,3_ = 8.6 Hz is in accord with an anti­periplanar arrangement of H2 and H3, whereas *J*_3,4_ = 4.6 Hz is consistent with the synclinal disposition of H3 and H4, resulting from a *gauche* arrangement for C2—C3—C4—C5.

The ^13^C NMR spectrum confirmed the presence of the four ring forms of d-ribose as indicated by their C1 signals and the major peak for C1 in the adduct at δ_C_ 82.25 was accompanied by a much smaller peak at δ_C_ 84.19 which suggests the presence in solution of the diastereoisomer of (1) having the *S*-configuration at C1.

## Supra­molecular features   

A three-dimensional network exists in the crystal structure through the coordination of each potassium cation (overall seven coordinate) to six different ribose bis­ulfite residues and through extensive hydrogen bonding between hy­droxy hydrogens and oxygen atoms of hydroxyl groups or those on sulfur. Although the addition of the sulfite anion to C1 of the ribose moiety can theoretically afford two isomers, only the *R*-diastereomer was present in the crystal studied.

## Synthesis, crystallization and spectroscopic analysis   

Water (0.5 ml) was added to potassium metabisulfite (0.37 g), which did not dissolve completely even on warming but which appeared to change its crystalline form as it underwent hydrolysis to yield potassium hydrogen sulfite. To this suspension was added a solution of d-ribose (0.5 g) in water (0.35 ml), leading to immediate and complete solution of the reaction mixture. Seed crystals were obtained by complete evaporation of a small proportion of the solution, and these were added to the bulk of the solution which was then stored at 277 K, leading to the formation of large, well-separated crystals. The syrupy nature of the mother liquor required its removal with a Pasteur pipette, after which the crystals were dried by pressing between filter papers, to give potassium (1*R*)-d-ribit-1-yl­sulfonate, m.p. 396–400 K (with decomposition); [α]_D_ −6.1 (15 min.) (*c*, 0.81 in 9:1 H_2_O:HOAc).

^1^H NMR (D_2_O, 400 MHz, reference *Me*_3_COH at δ_H_ 1.24): δ 5.37 (*d*, *J*_1,2_ = 3.8 Hz, H-1 of α-furan­ose), 5.24 (*d*, *J*_1,2_ = 1.8 Hz, H-1 of β-furan­ose), 4.92 (*d*, *J*_1,2_ = 6.5 Hz, H-1 of β-pyran­ose), 4.85 (*d*, *J*_1,2_ = 1.8 Hz, H-1 of α-pyran­ose); signals for acyclic sulfonate: δ_H_ 4.67 (*s*, H-1), 4.18 (*d*, *J*_2,3_ = 8.6 Hz, H-2), 3.94 (*ddd*, *J*_3,4_ = 4.6, *J*_4,5a_ = 3.1, *J*_4,5b_ = 7.4 Hz, H-4), 3.82 (*dd*, *J*_5a,5b_ = −11.9 Hz, H-5a), 3.77 (*dd*, H-3), 3.69 (*dd*, H-5b). ^13^C NMR (D_2_O, 100 MHz, reference *Me*_3_COH at δ_C_ 30.29): δ 101.55 (C1, β-furan­ose), 96.89 (C1, α-furan­ose), 94.43 (C1, β-pyran­ose), 94.15 (C1, α-pyran­ose); signals for adduct: 82.25 (C1), 73.23, 71.88, 70.61 (C2, C3, C4), 62.56 (C5). A small but significant peak was observed at δ_C_ 84.19.

Integration of the various signals for H-1 in the ^1^H NMR spectrum, 5 minutes after sample dissolution, indicated the species α-furan­ose, β-furan­ose, β-pyran­ose, α-pyran­ose, bis­ulfite adduct were present in the % ratios of 3.6:6.2:10.9:5.1:74.2. Re-measurement after 18 days, gave these % ratios as 1.5:2.6:16.2:8.7:70.9.

HRESMS (negative-ion mode, measured in H_2_O/MeOH, solution) gave an expected peak at *m*/*z* 231.0187 ([C_5_H_11_O_8_S]^−^), the base peak at 213.0082 ([C_5_H_11_O_8_S−H_2_O]^−^) and a significant peak at 299.0987 ([C_10_H_19_O_10_]^−^). The last peak corresponds to the ion of the product formed by reaction between the bis­ulfite adduct and d-ribose with displacement of potassium bis­ulfite.

## Refinement   

Crystal data, data collection and structure refinement details are summarized in Table 2 ▶. Hydrogen atoms bound to the carbon atoms were included in idealized positions (with C—H distances of 0.98 and 0.97 Å for methyne and methylene groups respectively) and their *U*_iso_ values were set to ride on the *U*_eq_ values of the parent atoms; hydroxyl hydrogen atoms were located in difference maps and were refined freely.

## Supplementary Material

Crystal structure: contains datablock(s) 1, New_Global_Publ_Block. DOI: 10.1107/S1600536814022685/sj5424sup1.cif

Structure factors: contains datablock(s) 1. DOI: 10.1107/S1600536814022685/sj54241sup2.hkl

Supporting information file. DOI: 10.1107/S1600536814022685/sj54241sup3.cml

CCDC reference: 1007337

Additional supporting information:  crystallographic information; 3D view; checkCIF report

## Figures and Tables

**Table 1:** Hydrogen-bond geometry (, )

*D*H*A*	*D*H	H*A*	*D* *A*	*D*H*A*
O1H1*O*O12^i^	0.83(3)	1.89(3)	2.6980(14)	165(2)
O2H2*O*O5^ii^	0.77(2)	2.34(3)	2.9111(14)	132(2)
O3H3*O*O2^iii^	0.79(2)	2.10(2)	2.8596(14)	162(2)
O4H4*O*O5^i^	0.83(3)	1.95(3)	2.7779(14)	175(3)
O5H5*O*O13^iv^	0.88(2)	1.99(2)	2.8432(14)	161.7(18)

Symmetry codes: (i) 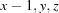; (ii) 
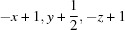
; (iii) 
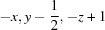
; (iv) 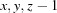.

**Table 2:** Experimental details

Crystal data	
Chemical formula	K^+^C_5_H_11_O_8_S
*M*_r_	270.30
Crystal system, space group	Monoclinic, *P*2_1_
Temperature (K)	140
*a*, *b*, *c* ()	5.36167(8), 9.01474(14), 9.78623(17)
()	102.8138(16)
*V* (^3^)	461.23(1)
*Z*	2
Radiation type	Mo *K*
(mm^1^)	0.83
Crystal size (mm)	0.22 0.22 0.12

Data collection	
Diffractometer	Oxford Diffraction Xcalibur 3/Sapphire3 CCD
Absorption correction	Multi-scan (*CrysAlis PRO*; Oxford Diffraction, 2011 ▶)
*T*_min_, *T*_max_	0.874, 1.00
No. of measured, independent and observed [*I* > 2(*I*)] reflections	8864, 2690, 2632
*R*_int_	0.023
(sin /)_max_ (^1^)	0.703

Refinement	
*R*[*F* ^2^ > 2(*F* ^2^)], *wR*(*F* ^2^), *S*	0.021, 0.053, 1.05
No. of reflections	2690
No. of parameters	156
No. of restraints	1
H-atom treatment	H atoms treated by a mixture of independent and constrained refinement
_max_, _min_ (e ^3^)	0.43, 0.22
Absolute structure	Flack (1983 ▶), 1264 Friedel pairs
Absolute structure parameter	0.01(3)

Computer programs: *CrysAlis PRO* (Oxford Diffraction, 2011 ▶), *SHELXS97* and *SHELXL97* (Sheldrick, 2008 ▶), *ORTEPII* (Johnson, 1976 ▶), *ORTEP-3 for Windows* and *WinGX* (Farrugia, 2012 ▶).

## supplementary crystallographic information

### Crystal data

**Table:**

K^+^·C_5_H_11_O_8_S^−^	*F*(000) = 280
*M**_r_* = 270.30	*D*_x_ = 1.946 Mg m^−^^3^
Monoclinic, *P*2_1_	Mo *K*α radiation, λ = 0.71073 Å
*a* = 5.36167 (8) Å	Cell parameters from 6553 reflections
*b* = 9.01474 (14) Å	θ = 3.1–32.4°
*c* = 9.78623 (17) Å	µ = 0.83 mm^−^^1^
β = 102.8138 (16)°	*T* = 140 K
*V* = 461.23 (1) Å^3^	Plate, colourless
*Z* = 2	0.22 × 0.22 × 0.12 mm

### Data collection

**Table:**

Oxford Diffraction Xcalibur 3/Sapphire3 CCD diffractometer	2690 independent reflections
Radiation source: Enhance (Mo) X-ray Source	2632 reflections with *I* > 2σ(*I*)
Graphite monochromator	*R*_int_ = 0.023
Detector resolution: 16.0050 pixels mm^-1^	θ_max_ = 30.0°, θ_min_ = 3.1°
Thin–slice φ and ω scans	*h* = −7→7
Absorption correction: multi-scan (*CrysAlis PRO*; Oxford Diffraction, 2011)	*k* = −12→12
*T*_min_ = 0.874, *T*_max_ = 1.00	*l* = −13→13
8864 measured reflections	

### Refinement

**Table:**

Refinement on *F*^2^	Secondary atom site location: difference Fourier map
Least-squares matrix: full	Hydrogen site location: inferred from neighbouring sites
*R*[*F*^2^ > 2σ(*F*^2^)] = 0.021	H atoms treated by a mixture of independent and constrained refinement
*wR*(*F*^2^) = 0.053	*w* = 1/[σ^2^(*F*_o_^2^) + (0.0318*P*)^2^ + 0.0276*P*] where *P* = (*F*_o_^2^ + 2*F*_c_^2^)/3
*S* = 1.05	(Δ/σ)_max_ < 0.001
2690 reflections	Δρ_max_ = 0.43 e Å^−^^3^
156 parameters	Δρ_min_ = −0.22 e Å^−^^3^
1 restraint	Absolute structure: Flack (1983), 1264 Friedel pairs
Primary atom site location: structure-invariant direct methods	Absolute structure parameter: −0.01 (3)

### Special details

**Table:**

Experimental. Absorption correction: CrysAlisPro RED, Oxford Diffraction Ltd., Version 1.171.33.55 Empirical absorption correction using spherical harmonics, implemented in SCALE3 ABSPACK scaling algorithm.
Geometry. All s.u.'s (except the s.u. in the dihedral angle between two l.s. planes) are estimated using the full covariance matrix. The cell s.u.'s are taken into account individually in the estimation of s.u.'s in distances, angles and torsion angles; correlations between s.u.'s in cell parameters are only used when they are defined by crystal symmetry. An approximate (isotropic) treatment of cell s.u.'s is used for estimating s.u.'s involving l.s. planes.
Refinement. Refinement of *F*^2^ against ALL reflections. The weighted *R*-factor *wR* and goodness of fit *S* are based on *F*^2^, conventional *R*-factors *R* are based on *F*, with *F* set to zero for negative *F*^2^. The threshold expression of *F*^2^ > 2σ(*F*^2^) is used only for calculating *R*-factors(gt) *etc*. and is not relevant to the choice of reflections for refinement. *R*-factors based on *F*^2^ are statistically about twice as large as those based on *F*, and *R*- factors based on ALL data will be even larger.

### Fractional atomic coordinates and isotropic or equivalent isotropic displacement parameters (Å^2^)

**Table:**

	*x*	*y*	*z*	*U*_iso_*/*U*_eq_	
K	0.00261 (5)	0.45609 (3)	−0.05055 (3)	0.01553 (7)	
C1	0.2210 (2)	0.19926 (14)	0.68818 (13)	0.0093 (2)	
H1	0.1956	0.0932	0.6679	0.011*	
C2	0.3088 (2)	0.27375 (14)	0.56579 (14)	0.0096 (2)	
H2	0.4902	0.2528	0.5714	0.012*	
C3	0.1458 (2)	0.21199 (15)	0.42818 (13)	0.0101 (2)	
H3	−0.0341	0.2168	0.4341	0.012*	
C4	0.1759 (2)	0.30205 (16)	0.30071 (14)	0.0114 (2)	
H4	0.1205	0.4036	0.3142	0.014*	
C5	0.4444 (2)	0.31169 (16)	0.27422 (14)	0.0120 (2)	
H5A	0.4418	0.3791	0.1967	0.014*	
H5B	0.5569	0.3539	0.3564	0.014*	
O1	−0.01482 (18)	0.26253 (12)	0.69708 (11)	0.0141 (2)	
O2	0.26721 (19)	0.42953 (10)	0.56585 (11)	0.01250 (19)	
O3	0.2107 (2)	0.05973 (11)	0.41598 (12)	0.0157 (2)	
O4	0.01062 (18)	0.24575 (13)	0.17548 (10)	0.0159 (2)	
O5	0.54900 (19)	0.17312 (11)	0.24301 (11)	0.01340 (19)	
S	0.44130 (5)	0.22060 (3)	0.85556 (3)	0.00909 (7)	
O11	0.48670 (19)	0.37852 (11)	0.87973 (11)	0.0165 (2)	
O12	0.67445 (18)	0.13911 (12)	0.84780 (11)	0.01367 (19)	
O13	0.30566 (18)	0.15410 (12)	0.95421 (11)	0.0155 (2)	
H1O	−0.095 (4)	0.211 (3)	0.743 (2)	0.031 (6)*	
H2O	0.388 (4)	0.464 (3)	0.611 (2)	0.030 (6)*	
H3O	0.087 (4)	0.010 (3)	0.410 (2)	0.027 (6)*	
H4O	−0.122 (5)	0.221 (3)	0.199 (3)	0.046 (7)*	
H5O	0.460 (4)	0.149 (2)	0.159 (2)	0.013 (4)*	

### Atomic displacement parameters (Å^2^)

**Table:**

	*U*^11^	*U*^22^	*U*^33^	*U*^12^	*U*^13^	*U*^23^
K	0.01277 (12)	0.01544 (13)	0.01879 (14)	0.00082 (11)	0.00440 (10)	−0.00328 (11)
C1	0.0088 (5)	0.0103 (6)	0.0087 (5)	−0.0003 (4)	0.0018 (4)	−0.0003 (4)
C2	0.0093 (5)	0.0087 (5)	0.0112 (6)	0.0001 (4)	0.0028 (4)	0.0016 (4)
C3	0.0091 (5)	0.0113 (6)	0.0100 (5)	0.0005 (4)	0.0027 (4)	−0.0003 (5)
C4	0.0106 (5)	0.0138 (6)	0.0098 (6)	0.0011 (4)	0.0023 (4)	−0.0016 (5)
C5	0.0105 (5)	0.0118 (6)	0.0139 (6)	−0.0003 (4)	0.0033 (4)	0.0003 (5)
O1	0.0078 (4)	0.0192 (5)	0.0157 (5)	0.0023 (3)	0.0037 (4)	0.0052 (4)
O2	0.0153 (4)	0.0079 (5)	0.0134 (4)	−0.0027 (3)	0.0014 (4)	0.0001 (3)
O3	0.0184 (5)	0.0084 (4)	0.0216 (5)	−0.0032 (4)	0.0076 (4)	−0.0020 (4)
O4	0.0092 (4)	0.0279 (6)	0.0102 (4)	−0.0016 (4)	0.0014 (3)	−0.0024 (4)
O5	0.0114 (4)	0.0154 (5)	0.0136 (5)	0.0017 (3)	0.0033 (4)	−0.0007 (4)
S	0.00795 (12)	0.01038 (14)	0.00897 (13)	−0.00016 (10)	0.00192 (9)	0.00056 (11)
O11	0.0176 (5)	0.0121 (5)	0.0182 (5)	−0.0016 (4)	0.0005 (4)	−0.0026 (4)
O12	0.0090 (4)	0.0165 (5)	0.0159 (5)	0.0024 (3)	0.0037 (3)	0.0031 (4)
O13	0.0133 (5)	0.0230 (5)	0.0112 (5)	−0.0024 (4)	0.0047 (4)	0.0029 (4)

### Geometric parameters (Å, º)

**Table:**

K—O13^i^	2.7383 (10)	C4—C5	1.5210 (17)
K—O11^ii^	2.7873 (10)	C4—H4	0.9800
K—O12^iii^	2.8519 (10)	C5—O5	1.4297 (17)
K—O4^iv^	2.8775 (12)	C5—H5A	0.9700
K—O4	2.9065 (11)	C5—H5B	0.9700
K—O11^v^	2.9115 (11)	O1—K^vi^	3.0085 (11)
K—O1^v^	3.0085 (11)	O1—H1O	0.83 (3)
K—O13^v^	3.1654 (11)	O2—H2O	0.77 (2)
K—O12^ii^	3.3874 (11)	O3—H3O	0.79 (2)
K—S^v^	3.4412 (4)	O4—K^vii^	2.8775 (12)
K—S^ii^	3.6306 (4)	O4—H4O	0.83 (3)
K—K^iv^	4.6161 (1)	O5—H5O	0.88 (2)
C1—O1	1.4071 (15)	S—O11	1.4547 (10)
C1—C2	1.5355 (17)	S—O13	1.4601 (10)
C1—S	1.8048 (13)	S—O12	1.4664 (10)
C1—H1	0.9800	S—K^vi^	3.4412 (4)
C2—O2	1.4220 (15)	S—K^viii^	3.6306 (4)
C2—C3	1.5380 (18)	O11—K^viii^	2.7873 (10)
C2—H2	0.9800	O11—K^vi^	2.9115 (10)
C3—O3	1.4275 (16)	O12—K^ix^	2.8519 (10)
C3—C4	1.5266 (18)	O12—K^viii^	3.3874 (11)
C3—H3	0.9800	O13—K^x^	2.7383 (10)
C4—O4	1.4364 (16)	O13—K^vi^	3.1654 (11)
			
O13^i^—K—O11^ii^	66.78 (3)	O1—C1—C2	107.84 (10)
O13^i^—K—O12^iii^	72.73 (3)	O1—C1—S	108.47 (9)
O11^ii^—K—O12^iii^	137.05 (3)	C2—C1—S	114.15 (8)
O13^i^—K—O4^iv^	66.10 (3)	O1—C1—H1	108.8
O11^ii^—K—O4^iv^	101.19 (3)	C2—C1—H1	108.8
O12^iii^—K—O4^iv^	74.01 (3)	S—C1—H1	108.8
O13^i^—K—O4	94.10 (3)	O2—C2—C1	110.87 (10)
O11^ii^—K—O4	82.47 (3)	O2—C2—C3	107.40 (10)
O12^iii^—K—O4	86.77 (3)	C1—C2—C3	108.16 (10)
O4^iv^—K—O4	155.53 (3)	O2—C2—H2	110.1
O13^i^—K—O11^v^	151.02 (3)	C1—C2—H2	110.1
O11^ii^—K—O11^v^	140.38 (4)	C3—C2—H2	110.1
O12^iii^—K—O11^v^	82.34 (3)	O3—C3—C4	111.78 (10)
O4^iv^—K—O11^v^	93.30 (3)	O3—C3—C2	108.63 (10)
O4—K—O11^v^	99.10 (3)	C4—C3—C2	112.36 (11)
O13^i^—K—O1^v^	138.55 (3)	O3—C3—H3	108.0
O11^ii^—K—O1^v^	78.76 (3)	C4—C3—H3	108.0
O12^iii^—K—O1^v^	144.12 (3)	C2—C3—H3	108.0
O4^iv^—K—O1^v^	100.61 (3)	O4—C4—C5	107.64 (10)
O4—K—O1^v^	103.82 (3)	O4—C4—C3	110.62 (11)
O11^v^—K—O1^v^	62.31 (3)	C5—C4—C3	116.45 (11)
O13^i^—K—O13^v^	154.51 (2)	O4—C4—H4	107.2
O11^ii^—K—O13^v^	105.44 (3)	C5—C4—H4	107.2
O12^iii^—K—O13^v^	104.88 (3)	C3—C4—H4	107.2
O4^iv^—K—O13^v^	138.67 (3)	O5—C5—C4	114.67 (11)
O4—K—O13^v^	60.45 (3)	O5—C5—H5A	108.6
O11^v^—K—O13^v^	46.81 (3)	C4—C5—H5A	108.6
O1^v^—K—O13^v^	55.64 (3)	O5—C5—H5B	108.6
O13^i^—K—O12^ii^	109.70 (3)	C4—C5—H5B	108.6
O11^ii^—K—O12^ii^	45.08 (3)	H5A—C5—H5B	107.6
O12^iii^—K—O12^ii^	152.63 (2)	C1—O1—K^vi^	114.91 (7)
O4^iv^—K—O12^ii^	132.73 (3)	C1—O1—H1O	112.8 (16)
O4—K—O12^ii^	65.93 (3)	K^vi^—O1—H1O	78.7 (17)
O11^v^—K—O12^ii^	99.24 (3)	C2—O2—H2O	106.9 (18)
O1^v^—K—O12^ii^	49.47 (3)	C3—O3—H3O	109.5 (17)
O13^v^—K—O12^ii^	60.68 (2)	C4—O4—K^vii^	129.46 (8)
O13^i^—K—S^v^	174.03 (2)	C4—O4—K	108.72 (8)
O11^ii^—K—S^v^	118.68 (2)	K^vii^—O4—K	105.89 (3)
O12^iii^—K—S^v^	101.37 (2)	C4—O4—H4O	105.4 (17)
O4^iv^—K—S^v^	113.59 (2)	K^vii^—O4—H4O	86.0 (19)
O4—K—S^v^	84.56 (2)	K—O4—H4O	121.3 (18)
O11^v^—K—S^v^	24.72 (2)	C5—O5—H5O	104.9 (12)
O1^v^—K—S^v^	47.283 (18)	O11—S—O13	112.59 (6)
O13^v^—K—S^v^	25.091 (18)	O11—S—O12	112.64 (6)
O12^ii^—K—S^v^	75.077 (17)	O13—S—O12	112.57 (6)
O13^i^—K—S^ii^	86.48 (2)	O11—S—C1	107.71 (6)
O11^ii^—K—S^ii^	21.47 (2)	O13—S—C1	103.57 (6)
O12^iii^—K—S^ii^	148.83 (2)	O12—S—C1	107.07 (6)
O4^iv^—K—S^ii^	118.82 (2)	O11—S—K^vi^	56.81 (4)
O4—K—S^ii^	71.50 (2)	O13—S—K^vi^	66.83 (4)
O11^v^—K—S^ii^	122.16 (2)	O12—S—K^vi^	164.56 (4)
O1^v^—K—S^ii^	65.17 (2)	C1—S—K^vi^	87.68 (4)
O13^v^—K—S^ii^	83.965 (19)	O11—S—K^viii^	44.54 (4)
O12^ii^—K—S^ii^	23.797 (17)	O13—S—K^viii^	125.40 (4)
S^v^—K—S^ii^	98.572 (10)	O12—S—K^viii^	68.76 (4)
O13^i^—K—K^iv^	42.00 (2)	C1—S—K^viii^	129.12 (4)
O11^ii^—K—K^iv^	104.16 (2)	K^vi^—S—K^viii^	98.572 (10)
O12^iii^—K—K^iv^	46.97 (2)	S—O11—K^viii^	113.98 (5)
O4^iv^—K—K^iv^	37.27 (2)	S—O11—K^vi^	98.48 (5)
O4—K—K^iv^	118.27 (2)	K^viii^—O11—K^vi^	140.38 (4)
O11^v^—K—K^iv^	109.44 (2)	S—O12—K^ix^	130.30 (6)
O1^v^—K—K^iv^	137.88 (2)	S—O12—K^viii^	87.44 (5)
O13^v^—K—K^iv^	149.78 (2)	K^ix^—O12—K^viii^	95.05 (3)
O12^ii^—K—K^iv^	149.228 (19)	S—O13—K^x^	157.30 (6)
S^v^—K—K^iv^	134.152 (11)	S—O13—K^vi^	88.08 (5)
S^ii^—K—K^iv^	125.527 (10)	K^x^—O13—K^vi^	102.63 (3)
			
O1—C1—C2—O2	42.80 (13)	S^v^—K—O4—K^vii^	−43.60 (2)
S—C1—C2—O2	−77.80 (11)	S^ii^—K—O4—K^vii^	57.34 (2)
O1—C1—C2—C3	−74.71 (12)	K^iv^—K—O4—K^vii^	178.39 (2)
S—C1—C2—C3	164.69 (8)	O1—C1—S—O11	−64.93 (10)
O2—C2—C3—O3	171.93 (10)	C2—C1—S—O11	55.31 (10)
C1—C2—C3—O3	−68.35 (12)	O1—C1—S—O13	54.54 (10)
O2—C2—C3—C4	47.73 (13)	C2—C1—S—O13	174.78 (9)
C1—C2—C3—C4	167.44 (10)	O1—C1—S—O12	173.69 (9)
O3—C3—C4—O4	60.64 (13)	C2—C1—S—O12	−66.06 (10)
C2—C3—C4—O4	−176.92 (10)	O1—C1—S—K^vi^	−10.95 (8)
O3—C3—C4—C5	−62.64 (15)	C2—C1—S—K^vi^	109.29 (9)
C2—C3—C4—C5	59.80 (15)	O1—C1—S—K^viii^	−110.12 (8)
O4—C4—C5—O5	−60.95 (15)	C2—C1—S—K^viii^	10.12 (11)
C3—C4—C5—O5	63.86 (15)	O13—S—O11—K^viii^	118.13 (6)
C2—C1—O1—K^vi^	−110.24 (9)	O12—S—O11—K^viii^	−10.48 (8)
S—C1—O1—K^vi^	13.85 (10)	C1—S—O11—K^viii^	−128.31 (6)
C5—C4—O4—K^vii^	67.79 (13)	K^vi^—S—O11—K^viii^	156.73 (7)
C3—C4—O4—K^vii^	−60.45 (13)	O13—S—O11—K^vi^	−38.60 (6)
C5—C4—O4—K	−63.02 (11)	O12—S—O11—K^vi^	−167.21 (5)
C3—C4—O4—K	168.74 (7)	C1—S—O11—K^vi^	74.95 (6)
O13^i^—K—O4—C4	−75.17 (8)	K^viii^—S—O11—K^vi^	−156.73 (7)
O11^ii^—K—O4—C4	−141.09 (8)	O11—S—O12—K^ix^	102.32 (8)
O12^iii^—K—O4—C4	−2.76 (8)	O13—S—O12—K^ix^	−26.30 (10)
O4^iv^—K—O4—C4	−40.59 (8)	C1—S—O12—K^ix^	−139.47 (7)
O11^v^—K—O4—C4	78.94 (8)	K^vi^—S—O12—K^ix^	58.2 (2)
O1^v^—K—O4—C4	142.50 (7)	K^viii^—S—O12—K^ix^	94.45 (7)
O13^v^—K—O4—C4	106.36 (8)	O11—S—O12—K^viii^	7.87 (6)
O12^ii^—K—O4—C4	175.06 (8)	O13—S—O12—K^viii^	−120.76 (5)
S^v^—K—O4—C4	98.99 (7)	C1—S—O12—K^viii^	126.08 (5)
S^ii^—K—O4—C4	−160.07 (8)	K^vi^—S—O12—K^viii^	−36.23 (17)
K^iv^—K—O4—C4	−39.02 (8)	O11—S—O13—K^x^	153.72 (15)
O13^i^—K—O4—K^vii^	142.24 (3)	O12—S—O13—K^x^	−77.63 (17)
O11^ii^—K—O4—K^vii^	76.33 (3)	C1—S—O13—K^x^	37.66 (17)
O12^iii^—K—O4—K^vii^	−145.35 (3)	K^vi^—S—O13—K^x^	119.12 (16)
O4^iv^—K—O4—K^vii^	176.82 (4)	K^viii^—S—O13—K^x^	−156.92 (13)
O11^v^—K—O4—K^vii^	−63.64 (3)	O11—S—O13—K^vi^	34.60 (6)
O1^v^—K—O4—K^vii^	−0.08 (3)	O12—S—O13—K^vi^	163.25 (5)
O13^v^—K—O4—K^vii^	−36.23 (3)	C1—S—O13—K^vi^	−81.46 (5)
O12^ii^—K—O4—K^vii^	32.47 (3)	K^viii^—S—O13—K^vi^	83.96 (4)

Symmetry codes: (i) −*x*, *y*+1/2, −*z*+1; (ii) *x*−1, *y*, *z*−1; (iii) −*x*+1, *y*+1/2, −*z*+1; (iv) −*x*, *y*+1/2, −*z*; (v) *x*, *y*, *z*−1; (vi) *x*, *y*, *z*+1; (vii) −*x*, *y*−1/2, −*z*; (viii) *x*+1, *y*, *z*+1; (ix) −*x*+1, *y*−1/2, −*z*+1; (x) −*x*, *y*−1/2, −*z*+1.

### Hydrogen-bond geometry (Å, º)

**Table:**

*D*—H···*A*	*D*—H	H···*A*	*D*···*A*	*D*—H···*A*
O1—H1*O*···O12^xi^	0.83 (3)	1.89 (3)	2.6980 (14)	165 (2)
O2—H2*O*···O5^iii^	0.77 (2)	2.34 (3)	2.9111 (14)	132 (2)
O3—H3*O*···O2^x^	0.79 (2)	2.10 (2)	2.8596 (14)	162 (2)
O4—H4*O*···O5^xi^	0.83 (3)	1.95 (3)	2.7779 (14)	175 (3)
O5—H5*O*···O13^v^	0.88 (2)	1.99 (2)	2.8432 (14)	161.7 (18)

Symmetry codes: (iii) −*x*+1, *y*+1/2, −*z*+1; (v) *x*, *y*, *z*−1; (x) −*x*, *y*−1/2, −*z*+1; (xi) *x*−1, *y*, *z*.
